# Research Progress on Protein–Polyphenol Interaction Mechanisms and Structure–Activity Relationship Modified by Physical Fields

**DOI:** 10.3390/foods15142431

**Published:** 2026-07-09

**Authors:** Xiangjun Hu, Chengcheng Liu, Yutang Wang, Xi Yang, Lijun Sun

**Affiliations:** 1 College of Food Science and Engineering, Northwest A&F University, Yangling 712100, China; xiangjunhu@nwafu.edu.cn (X.H.); 18845797390@163.com (C.L.); wyt991023@nwsuaf.edu.cn (Y.W.); 2 College of Food Science and Engineering, Ningbo University, Ningbo 315000, China

**Keywords:** physical techniques, proteins, polyphenols, interactions, structure–activity relationships, functional properties, food applications

## Abstract

Due to limitations in stability, solubility, and functional properties, natural proteins often fail to meet the demands of industrial production. Traditional chemical or enzymatic methods for protein modification suffer from high costs and the generation of numerous byproducts. In contrast, physical techniques offer a new approach to the green modification of macromolecules by inducing structural changes in proteins. Based on these techniques, the construction of protein–polyphenol composite systems is considered an effective strategy for enhancing food quality. This article systematically reviews the molecular mechanisms by which physical fields regulate protein–polyphenol interactions, summarizes how physical techniques improve the functional properties of composite systems and their applications in food, and aims to provide theoretical support for the development of novel functional food ingredients and their industrial applications.

## 1. Introduction

As essential components within food matrices, proteins and polyphenols interact in ways that significantly enhance the physicochemical and functional qualities of food products [1]. Natural proteins are rich in various hydrophilic and hydrophobic residues, making them excellent emulsifiers and gelling agents, while polyphenols possess good antioxidant properties and bioactivity. However, the practical application of unbound polyphenols remains a challenge. They rapidly degrade under standard commercial processing and storage conditions—such as exposure to oxygen, elevated temperatures, and light—as well as within the harsh environment of the gastrointestinal tract, which severely diminishes their bioaccessibility [2]. Therefore, combining proteins with polyphenols to form composite systems is considered an effective method for protecting polyphenols from environmental stress and achieving targeted delivery [3].

However, in aqueous systems, natural protein molecules typically exhibit highly compact folded structures and tend to self-assemble into aggregates via non-covalent bonds, which limits the effective binding of exogenous polyphenols to proteins [4]. To address this issue, the traditional industrial approach typically employs strong bases, transglutaminase treatment, glycation (Maillard reaction), thermal processing, or enzymatic catalysis to promote the formation of covalent bonds. However, these traditional methods have significant limitations. For instance, strong bases can lead to amino acid degradation and nutrient loss, while enzymatic methods face issues such as high substrate specificity, difficulty in controlling the reaction endpoint, and high costs [5]. Therefore, the search for a new, efficient, green, and controllable method for macromolecular modification has become an urgent priority in the field of food colloids.

In recent years, physical technologies such as ultrasound, high-pressure homogenization, and cold plasma have provided new avenues for overcoming the bottlenecks of traditional macromolecular modification techniques. Their core mechanism involves introducing energy through cavitation effects, mechanical shearing, or high-energy particles to disrupt protein aggregates, induce conformational changes, and expose internal hydrophobic regions and free nucleophilic groups, thereby promoting their interaction with polyphenols [6]. More importantly, certain physical techniques can also induce the generation of reactive oxygen species from water molecules. Without the introduction of chemical cross-linking agents, these techniques can efficiently drive the oxidation of polyphenols and subsequent nucleophilic addition reactions, thereby binding physically modified proteins to polyphenols and significantly enhancing the interfacial activity, antioxidant capacity, and gel network strength of the composite system [7]. This article provides a systematic review of the mechanisms and structure–activity relationships underlying the regulation of protein–polyphenol interactions by physical fields, aiming to supply a theoretical foundation for sustainable food processing and the creation of functional ingredients.

Although several recent reviews have discussed protein–polyphenol interactions and related processing strategies, this review is distinguished by its focus on the role of physical fields in regulating these interactions. Rather than treating physical processing as a simple pretreatment step, we emphasize how different technologies modulate protein conformation, interaction mechanisms, and functional performance. In particular, this review provides a comparative analysis of representative physical techniques and highlights their distinct effects on the formation and properties of protein–polyphenol complexes. By integrating mechanism, functionality, and practical relevance, this review aims to offer a more focused perspective on the physical field-assisted design of protein–polyphenol systems.

## 2. The Effect of Physical Technologies on Protein Structure

The basic principle of physical techniques acting on proteins is to induce conformational changes by introducing external energy; this energy overcomes the resistance caused by the tightly folded protein structure, inducing protein aggregates to undergo unfolding, partial dissociation, or rearrangement into a more open and flexible conformation. As the spatial structure of protein gradually unfolds, hydrophobic regions and active groups originally hidden within the molecule are released. This not only reduces the resistance to polyphenol molecules approaching the protein but also provides sufficient space and reaction sites for subsequent protein–polyphenol binding.

### 2.1. Aggregated State

The energy input from physical fields first acts on the protein aggregation network, inducing significant depolymerization of macroscopic particles. Taking mechanical fields as an example, research by Cheng et al. showed that after dynamic high-pressure microjet treatment, the hydrated particle size of oat protein isolate decreased by 70.96%, while the absolute value of the zeta potential increased by 33.51%. This indicates that macromolecular aggregates were dispersed into uniform particles under the action of shear forces [8]. Similarly, Lee et al. confirmed that high-pressure homogenization could drastically reduce the average particle size of porcine myofibrillar proteins from 886 nm to 172 nm. High-molecular-weight myosin aggregates were effectively disaggregated by high-pressure shear forces, forming a highly dispersed system [9]. In addition to mechanical force fields, electromagnetic and non-thermal physical fields have also demonstrated excellent depolymerization effects. Short-duration (less than 30 s) microwave irradiation can rapidly open the compact structure of oxidized soy protein, suppressing its tendency to re-aggregate by reducing the cross-linking of intermolecular disulfide bonds [10]. Studies on cold plasma treatment of chickpea isolated protein and bovine myofibrillar protein indicate that high-energy active particles can introduce more polar groups onto the surfaces of macromolecules. Enhanced electrostatic repulsion forces the original aggregation network to disintegrate, thereby leading to a reduction in particle size and a homogenization of the distribution [11,12]. As aggregates dissociate, the protein dispersion system gradually transitions from a thermodynamically unstable suspension phase to a kinetically stable nanodispersed phase. This evolution of the aggregate structure lays the foundation for subsequent penetration of physical field energy into the molecular backbone, inducing changes in secondary, tertiary, and quaternary structures.

### 2.2. Secondary Structure

The secondary structure of proteins primarily consists of α-helices, β-sheets, β-turns, and random coils. The energy input from physical fields can effectively disrupt the hydrogen bond networks that maintain these secondary structures, thereby inducing the transformation of polypeptide chains from a highly ordered, compact state to a relatively loose and flexible conformation. Extensive research indicates that different physical fields play a significant role in reshaping protein secondary structures. For example, Liang et al. found that after high-pressure homogenization, intense mechanical shear forces disrupted the intermolecular hydrogen bonds in soybean concentrate. Infrared spectroscopy revealed a decrease in β-sheet content and a significant increase in random coil structure. Similarly, Wang et al. discovered that dynamic ultra-high-pressure homogenization could disrupt the stable hydrogen bond network of whey protein isolate, leading to a substantial reduction in α-helix content and causing protein denaturation [13]. In addition to mechanical fields, electromagnetic fields have also demonstrated significant regulatory effects: Wang et al. noted in their study of quinoa protein isolate that appropriate microwave treatment induces the conversion of α-helices to β-sheets, thereby disrupting the original compact secondary structure and rendering the overall protein conformation more loosely organized [14]. Furthermore, non-thermal physical fields, such as high-pressure atmospheric cold plasma, also possess powerful modification capabilities. Xu et al. found that after 60 min of treatment, the dense α-helix structure of bovine serum albumin decreased by 27%, while random coiling increased by 10%, and the polypeptide chain exhibited distinct denaturation behavior [15]. This physical-field-induced transition from a compact secondary structure to a loose conformation, such as random coiling, effectively reduces the steric hindrance caused by the compact folding of the polypeptide chain. Consequently, the active groups within the hydrophobic core are exposed, providing not only a “channel” for free polyphenol molecules to enter the protein interior, but also creating a critical conformational foundation for subsequent efficient noncovalent or covalent binding within the complex system [16].

### 2.3. Tertiary and Quaternary Structures

The tertiary and quaternary spatial structures of proteins are primarily maintained by noncovalent interactions such as hydrophobic interactions, hydrogen bonds, electrostatic forces, and van der Waals forces. Additionally, some proteins can further stabilize their structures through disulfide bonds (-S-S-). Under the influence of physical fields, the unfolding of the tertiary hydrophobic core and the dissociation of quaternary subunits typically occur simultaneously as synergistic thermodynamic processes. The introduction of physical field energy disrupts the original three-dimensional spatial network equilibrium, exposing the hydrophobic core and inducing changes in the microenvironment of amino acid residues as well as disulfide bond rearrangement. This is specifically manifested as changes in surface hydrophobicity (H_0_) and the content of free sulfhydryl groups (–SH) [17]. Studies have shown that ultra-high pressure can effectively disrupt the non-covalent bonds that maintain the tertiary structure within plant proteins such as soybean, causing the originally buried hydrophobic regions to flip toward the protein–water interface, resulting in a significant increase in the surface hydrophobicity of the system [18]. In addition to mechanical force fields, non-thermal electromagnetic fields can also disrupt this spatial conformational equilibrium: taking pulsed electric fields as an example, brief high-energy electric field pulses can cause charge rearrangement of polar groups within proteins; studies on whey protein isolates and various plant proteins have shown that pulsed electric fields alter the polarity of the microenvironment surrounding tryptophan residues, thereby inducing protein denaturation and significantly increasing surface hydrophobicity [19].

Furthermore, physical fields can effectively disrupt the covalent cross-linking networks that maintain compact spatial conformations, thereby exposing internal sulfur-containing groups. For instance, in a beef myofibrillar protein system, the cavitation effect of ultrasound disrupted the original tertiary structure, releasing free thiol groups from the core into the surface microenvironment, resulting in a marked increase in both free thiol content and surface hydrophobicity [20]. Similarly, dynamic high-pressure microjets (DHPM), which possess extremely strong physical disruption capabilities, also led to significant unfolding of compact conformations and extensive exposure of free thiol groups when treating Perilla frutescens isolated proteins at a pressure of 120 MPa [21]. These changes in microstructure directly determine the subsequent binding pathways between proteins and polyphenols. In non-covalent binding systems, the externalization of aromatic amino acids and hydrophobic residues effectively reduces steric hindrance, providing sufficient hydrophobic binding sites for polyphenol molecules and thereby significantly enhancing the interactions between proteins and polyphenols [22]. In covalent binding systems, the large number of exposed free thiol groups exhibit strong nucleophilicity. Studies have shown that when polyphenol molecules in the system are excited by a physical field or under basic conditions and oxidized to electrophilic quinone or semiquinone radical intermediates, the accumulated free sulfhydryl groups can act as nucleophiles and undergo irreversible cross-linking reactions with quinone compounds via nucleophilic reaction pathways such as the Michael addition. This reaction is a key step in promoting the formation of covalent bonds between proteins and polyphenols [2].

It is noteworthy that the extent and nature of structural changes vary considerably across different physical technologies. Ultrasonic treatment primarily induces cavitation and local heating, which preferentially disrupts hydrophobic interactions and hydrogen bonds, whereas high-pressure homogenization and dynamic high-pressure microfluidization exert strong mechanical shear forces that more effectively break down large aggregates. Pulsed electric fields mainly affect the electrostatic microenvironment through charge rearrangement, microwave provides rapid volumetric heating, and cold plasma generates reactive species that simultaneously modify protein structure through oxidative pathways. This mechanistic diversity suggests that the choice of physical technology should be tailored to the target protein characteristics and desired functional outcomes. A comparative overview of the advantages, limitations, and industrial scalability of these technologies is presented in Table 1.

## 3. Mechanisms and Characterization of Protein–Polyphenol Interactions Modified by Physical Fields

**Figure 1:**
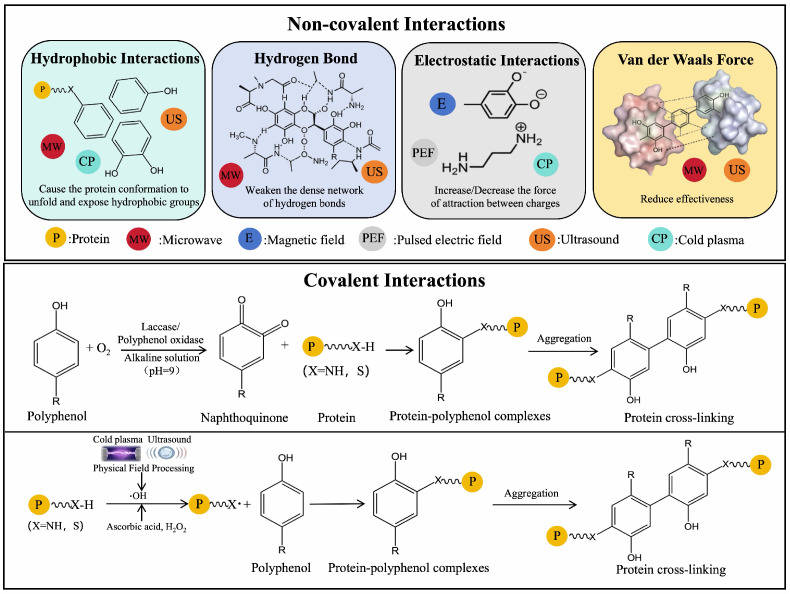
Mechanism of protein–polyphenol interaction regulated by physical fields.

The core of physical field-mediated regulation of protein–polyphenol interactions lies in altering the spatial structure of proteins through the input of external energy, thereby creating more favorable conditions for polyphenol binding. Depending on the intensity of the physical field energy and the reaction microenvironment, there are two primary modes of binding: non-covalent interactions and covalent interactions as shown in Figure 1. Non-covalent interactions typically occur after moderate protein denaturation, at which point polyphenol molecules primarily bind to newly exposed binding sites through hydrophobic interactions and hydrogen bonds. However, when the physical field energy is high, or when the system is induced to generate a large number of reactive free radicals, polyphenol molecules are prone to oxidation, leading to irreversible chemical reactions with reactive amino acid groups on the protein and forming more stable covalent bonds.

### 3.1. Noncovalent Interactions

Noncovalent interactions represent the most common binding mode between proteins and polyphenols, primarily involving hydrophobic interactions, hydrogen bonds, electrostatic interactions, and van der Waals forces. Such binding is typically reversible [37], and physical techniques can induce moderate protein denaturation, thereby creating conditions for noncovalent binding with polyphenols. For example, the cavitation effect and strong mechanical shear forces generated by ultrasound can effectively disrupt the hydrogen bonds and hydrophobic interactions that maintain the protein’s spatial structure [22]. Spectroscopic techniques such as circular dichroism (CD) and Fourier-transform infrared spectroscopy (FTIR) have consistently demonstrated that, following physical treatment, the content of α-helices in proteins typically decreases, shifting toward β-sheets and random coils. This conformational change exposes hydrophobic amino acid residues, such as tryptophan and tyrosine, which were originally hidden within the molecule, to the aqueous environment [35]. Upon protein denaturation, polyphenol molecules bind to the newly exposed hydrophobic sites of the protein via their aromatic ring structures, forming stable complexes primarily through hydrophobic interactions. Fluorescence spectroscopy analysis indicates that this interaction manifests as a static quenching process, and the interaction between whey protein and anthocyanins is stronger after moderate heat treatment than in untreated native protein [21]. Computational molecular docking techniques have also confirmed this. For example, under the influence of a pulsed electric field, pea proteins exposed more active regions, leading to a significant increase in the number of effective binding sites for epigallocatechin gallate [29]. Furthermore, isothermal titration calorimetry (ITC) and calculations of thermodynamic parameters have further revealed the underlying driving forces of non-covalent binding. Multiple studies have shown that during the physical field-mediated binding of proteins to polyphenols, the change in Gibbs free energy is typically negative (ΔG < 0), indicating that the binding process is spontaneous. When proteins interact with polyphenols, the entropy of the system generally decreases (ΔS < 0), suggesting that hydrogen bonds and van der Waals forces are the primary driving forces. Concurrently, the phenolic hydroxyl groups in polyphenol molecules can form dense hydrogen bonds with polar groups on the protein surface. This exothermic reaction (ΔH < 0) further stabilizes the spatial structure of the non-covalent complex [38,39].

### 3.2. Covalent Interactions

Unlike non-covalent binding, covalent binding between proteins and polyphenols is typically irreversible. This type of binding confers strong mechanical strength and stability to the complex, making it suitable for applications in food processing [40,41]. In traditional food macromolecule modification systems, covalent bonding methods include alkali treatment (pH 9), enzymatic methods (e.g., polyphenol oxidase or laccase), and radical-mediated methods (e.g., ascorbic acid and hydrogen peroxide) to catalyze the reaction [42]. The introduction of physical fields not only avoids the residual presence of chemical reagents but also provides a highly efficient and environmentally friendly new radical reaction pathway for covalent bonding. For example, during cold plasma or ultrasonic treatment, the mechanisms of hydroxyl radical generation differ fundamentally between technologies. In cold plasma systems, high-energy electrons generated by electrical discharge collide with water molecules, efficiently producing abundant hydroxyl radicals through dissociative excitation at atmospheric pressure and near-ambient temperature [32]. In ultrasonic systems, hydroxyl radicals are generated through a different route: acoustic cavitation creates localized hot spots with transient high temperatures that can dissociate water molecules; however, the radical yield is considerably lower than in plasma and is highly dependent on ultrasonic frequency, power density, and dissolved gas content [6,24]. These radicals can rapidly abstract hydrogen atoms from the phenolic hydroxyl groups on the aromatic rings of free polyphenols, oxidizing them into quinone derivatives; meanwhile, the aromatic carbon rings in the quinone structures act as excellent electrophiles, binding to electron-rich nucleophilic groups on protein side chains to form complexes. Importantly, the efficiency of this oxidation process is governed by multiple interrelated parameters specific to each technology. For cold plasma, EGCG oxidation to quinones proceeds within 45 s of treatment, achieving covalent conjugation efficiencies comparable to conventional alkaline treatment [32]. For ultrasound-assisted systems, the oxidation of rutin in whey protein isolate complexes increased with power density in the range of 200–600 W over 10–30 min [24], and similar power- and time-dependent enhancement was observed for EGCG conjugation with walnut protein isolate under ultrasonic treatment [43]. These examples demonstrate that the dominant oxidation pathway and its efficiency are strongly influenced by the specific physical field type, treatment intensity, exposure time, and matrix composition. This process is primarily achieved through two classic organic chemical reaction mechanisms: the first is the Michael addition reaction, in which quinone molecules undergo a nucleophilic addition reaction directly with the thiol group of cysteine or the amino group of lysine in proteins, forming stable C–N or C–S single bonds. The second is the Schiff base reaction, in which the carbonyl group of the oxidized polyphenol undergoes a dehydration condensation reaction with the primary amine group on the protein, generating cross-linked products containing a C=N double bond [23]. Studies have shown that after ultrasonic treatment, the content of free thiol groups and free amino groups in the whey protein isolate–rutin complex system was significantly reduced, indicating that these key nucleophilic groups had been largely consumed by the quinones generated by the oxidation of rutin, forming covalent bonds [24]. Similarly, in a complex system composed of walnut protein isolate and epigallocatechin gallate, ultrasonic treatment not only accelerated the formation of covalent bonds but also significantly altered the three-dimensional structure of the protein, leading to an increase in its absolute zeta potential and thereby enhancing its emulsifying properties [43]. Furthermore, studies have shown that the binding efficiency of β-lactoglobulin with epigallocatechin gallate after 45 s of cold plasma treatment is comparable to that of alkaline treatment and significantly higher than that of traditional radical-mediated methods (ascorbic acid and H_2_O_2_), while the antioxidant activity, emulsifying properties, and digestive sensitization of the complex are also improved [32].

When comparing the non-covalent and covalent binding pathways, it is evident that the distinction between these two modes is not always absolute; moderate treatment intensities may simultaneously promote both binding types, and the relative contribution of each depends on specific physical field parameters, reaction time, and polyphenol-to-protein ratio. Future studies should systematically map these transition boundaries to enable more precise control over the binding outcome.

### 3.3. Characterization Techniques

**Figure 2:**
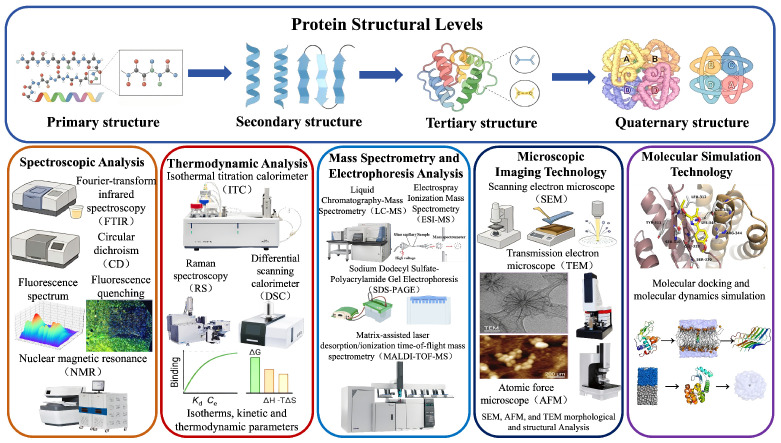
Multilevel structure of proteins and complex characterization techniques.

To accurately elucidate the patterns of protein conformation evolution and binding mechanisms under the influence of physical fields, modern food chemistry extensively employs multi-scale, multi-dimensional characterization techniques as shown in Figure 2. First, spectroscopic and thermodynamic methods are used to preliminarily determine the nature of the interactions and the influence of conformation; second, appropriate mass spectrometry and electrophoresis techniques are selected to analyze the molecular level; finally, microscopic techniques are employed to observe macroscopic aggregation behavior. Together, these steps form a comprehensive chain of evidence revealing the interactions between proteins and polyphenols.

In addition to these well-established techniques, several emerging characterization approaches are gaining prominence in the study of protein–polyphenol interactions. Small-angle X-ray scattering (SAXS) has been employed to investigate the solution structure of protein–polyphenol complexes; for instance, Shi et al. used SAXS to reveal that tea polyphenols induce distinct conformational changes in bovine serum albumin and β-lactoglobulin, providing low-resolution structural information on the overall shape and compactness of the resulting complexes [44]. Cryo-electron microscopy (cryo-EM) has been applied to visualize polyphenol-induced protein assembly; Xu et al. recently demonstrated that EGCG inactivates tumor necrosis factor-alpha (TNFα) by inducing its higher-order oligomeric assembly as resolved by cryo-EM [45]. Native mass spectrometry (native MS) preserves non-covalent interactions during ionization, enabling direct determination of binding stoichiometry; Alexander et al. used native MS to characterize the complexes of human and bovine serum albumins with various flavonoids, revealing binding stoichiometries and relative binding affinities [46]. Hydrogen–deuterium exchange mass spectrometry (HDX-MS) offers residue-level insights into conformational changes and binding interfaces; Grønnemose et al. applied HDX-MS to elucidate the dual effects of EGCG on α-synuclein oligomerization, identifying specific regions affected by polyphenol binding [47]. The integration of these complementary techniques promises a more comprehensive understanding of the structural basis underlying protein–polyphenol interactions.

#### 3.3.1. Secondary Structure

Interactions between proteins and polyphenols can trigger conformational rearrangements of the polypeptide chain. At the secondary structure level, spectroscopic techniques provide non-destructive and highly sensitive detection methods, among which Fourier-transform infrared spectroscopy (FTIR) and circular dichroism (CD) are frequently used in combination. Specifically, FTIR primarily evaluates the hydrogen-bond network maintaining the secondary structure through semi-quantitative analysis of the characteristic amide I band (1600–1700 cm^−1^) in the polypeptide backbone [48]. Circular dichroism (CD) spectroscopy, on the other hand, quantitatively analyzes changes in the content of protein secondary structures following interactions by measuring spectral differences in the far-ultraviolet region, thereby intuitively reflecting changes in the proportion of conformational transitions—such as from α-helices to β-sheets or random coils—after the introduction of polyphenols [49]. Furthermore, Raman spectroscopy (RS), as an important complementary technique, not only analyzes the Amide I band but also reveals the mechanisms of non-covalent interactions such as hydrogen bonding through characteristic vibrational peaks of specific residues like tyrosine, while detecting disulfide bond cleavage and changes in the microenvironment of amino acid side chains [50].

To provide a concrete illustration, FTIR and CD analyses of non-covalent complexes formed between lactoferrin (LaF) and theaflavin (TF) revealed significant secondary structure rearrangements. The amide I band of LaF shifted from 1637.3 cm^−1^ to 1635.4, 1633.3, and 1629.6 cm^−1^ with increasing TF concentration, indicating progressive protein unfolding. CD spectral deconvolution showed that the secondary structure of native LaF comprised α-helix (7.40 ± 0.44%), β-sheet (38.73 ± 0.78%), β-turns (21.76 ± 0.35%), and unordered structures (32.10 ± 0.63%). Upon TF binding, the α-helix and β-turn contents significantly decreased (*p* < 0.05), while the β-sheet content gradually increased from 38.73 ± 0.78% to 42.30 ± 0.57% (*p* < 0.05), and the unordered fraction increased, collectively reflecting a TF-induced conformational transition toward a more open and flexible structure. Molecular docking further confirmed that the stable binding was driven by hydrogen bonding and hydrophobic interactions [51]. Similarly, comparative FTIR studies of soy protein isolate (SPI) complexed with two isomeric polyphenols, quercetin and morin (differing only in the B-ring hydroxyl group positions), demonstrated that both induced the conversion of β-sheet to disordered structures, but quercetin—with its 3′,4′-dihydroxyl configuration—exhibited stronger binding affinity to SPI (−8.5 kcal/mol for 7S fraction) than morin with 2′,4′-hydroxyl groups (−8.1 kcal/mol), as determined by molecular docking [52].

#### 3.3.2. Tertiary Structure

**Figure 3:**
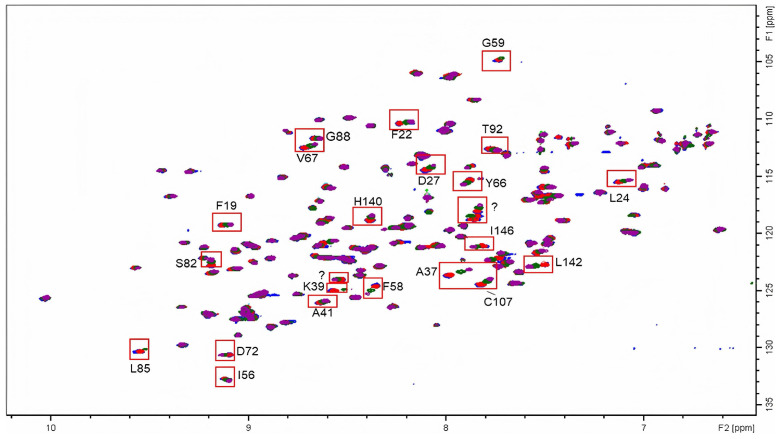
Overlay of 1H–15N-HSQC NMR spectra of the backbone amide protons of 100 μM uniformly labeled r-Mal d 1.01 (blue) in the presence of 0.1 mM (red), 1 mM (green), and 2 mM (violet) chlorogenic acid. Red boxes highlight cross-peaks with remarkable chemical shift changes induced by chlorogenic acid, and each annotated number corresponds to the perturbed amino acid residue. Unassignable signals are labelled with a question mark [55].

Regarding changes in the three-dimensional folding state of proteins, intrinsic fluorescence spectroscopy is a core technique for characterizing the microenvironment of aromatic amino acids. When polyphenol molecules enter the hydrophobic core of a protein or bind to the protein surface, they typically cause fluorescence quenching of tryptophan or tyrosine residues, accompanied by a red shift or blue shift in the maximum emission wavelength. By fitting the quenching data using the Stern–Volmer equation, one can accurately distinguish between dynamic and static quenching mechanisms, thereby calculating the binding constant (Ka) and the number of binding sites (*n*), which provides a thermodynamic basis for evaluating binding affinity. To analyze the structure in greater detail, synchronous fluorescence spectroscopy can capture changes in the microenvironments of tryptophan and tyrosine by altering the excitation wavelength; meanwhile, three-dimensional fluorescence spectroscopy can reflect changes in the protein polypeptide chain backbone structure while obtaining information on the microenvironments of individual residues, thereby enabling multidimensional structural characterization of the complex system [53]. After elucidating the overall changes in the tertiary structure, more precise analytical methods are typically required to further investigate specific binding sites and energy drivers. Nuclear magnetic resonance (NMR) technology, by monitoring changes in the chemical shifts in specific protons, can identify the specific amino acid residue sequences involved in binding to polyphenols at the atomic level [54]. Kaeswurm et al. characterized the complex of the apple allergen protein (Mal d 1) with chlorogenic acid using high-resolution two-dimensional 1H-15N heteronuclear single-quantum coherent (HSQC) spectroscopy as shown in Figure 3. They performed a spectral overlay comparison between the spectrum of the free protein and that of the complex formed after the addition of polyphenols. The results showed significant shifts in chemical shift or signal intensity attenuation at specific cross-peaks in the spectrum. By further isolating these cross-peaks exhibiting characteristic changes and mapping them to the protein’s primary sequence, the specific amino acid residues involved in non-covalent interactions with the polyphenol can be identified [55].

**Figure 4:**
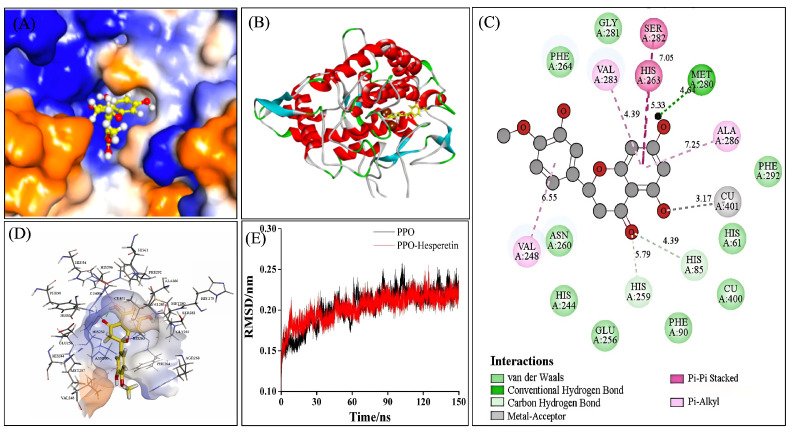
Binding mode of hesperetin with polyphenol oxidase (PPO): (**A**) Hydrophobic binding of hesperetin to PPO. Blue and orange represent the hydrophilic and hydrophobic parts of the enzyme surface, respectively. (**B**) 3D view of hesperetin bound to PPO. (**C**) Amino acid residues of PPO interacting with hesperetin (2D schematic interaction diagram). (**D**) Hydrophobic surface and surrounding amino acid residues in the binding area of hesperetin to PPO. (**E**) RMSD of PPO and the PPO–hesperetin complex from a 150 ns molecular dynamics simulation of PPO with hesperetin [60].

In terms of energy driving, isothermal titration calorimetry (ITC) and differential scanning calorimetry (DSC) provide direct thermodynamic evidence. By precisely quantifying the changes in enthalpy, entropy, and Gibbs free energy during titration, ITC not only confirms the spontaneity of binding but also determines the dominant type of binding interaction [56]; DSC, on the other hand, intuitively reflects the impact of polyphenol incorporation on the thermal stability of the protein’s overall spatial network by monitoring shifts in the denaturation temperature (Td) [57]. Combining molecular docking and molecular dynamics simulations not only visually demonstrates the dynamic binding of polyphenols to the tertiary structure of protein in a virtual space, but also extracts parameters such as the root-mean-square deviation (RMSD) to dynamically assess the spatial stability of the complex, thereby complementing and validating spectroscopic and thermodynamic data [58,59]. In studying the interaction mechanism between naringin and polyphenol oxidase, molecular docking was employed to elucidate the hydrogen bond network and bond lengths formed between the hydroxyl group of polyphenol and specific protein amino acid residues (e.g., Met280 and His89). Subsequently, the core parameter of root-mean-square deviation (RMSD) was extracted from molecular dynamics simulations spanning tens of nanoseconds to dynamically quantify the structural fluctuations of the complex. The analysis revealed that the RMSD curve of the protein in the bound state reached a plateau significantly earlier than that of the free state, and the overall fluctuation values were markedly reduced. This indicates that the binding of polyphenol molecules effectively restricts the flexible extension of the polypeptide chains and enhances the spatial rigidity and stability of the protein’s three-dimensional framework, consistent with the conformational changes observed in fluorescence spectroscopy and circular dichroism spectroscopy as shown in Figure 4 [60].

As a case in point, thermodynamic analysis of the interaction between bovine lactoferrin (BLF) and chlorogenic acid (CA) using fluorescence spectroscopy at multiple temperatures (290 K and 300 K) revealed spontaneous binding. The binding constants (Ka) were determined to be 5.29 × 10^3^ L mol^−1^ at 290 K and 5.19 × 10^3^ L mol^−1^ at 300 K, with the corresponding Gibbs free energy changes (ΔG) of −26.23 kJ mol^−1^ and −27.09 kJ mol^−1^, respectively, confirming spontaneity. The negative enthalpy change (ΔH = −1.35 kJ mol^−1^ at 300 K) combined with a positive entropy change (ΔS = 85.79 J mol^−1^ K^−1^) indicated that both hydrogen bonding/van der Waals forces and hydrophobic interactions contributed to the binding process. FTIR analysis further showed that the amide I band of BLF shifted from 1648.8 cm^−1^ in the native protein after CA addition, confirming secondary structure changes upon complexation [5].

#### 3.3.3. Quaternary Structure and Aggregation State

**Figure 5:**
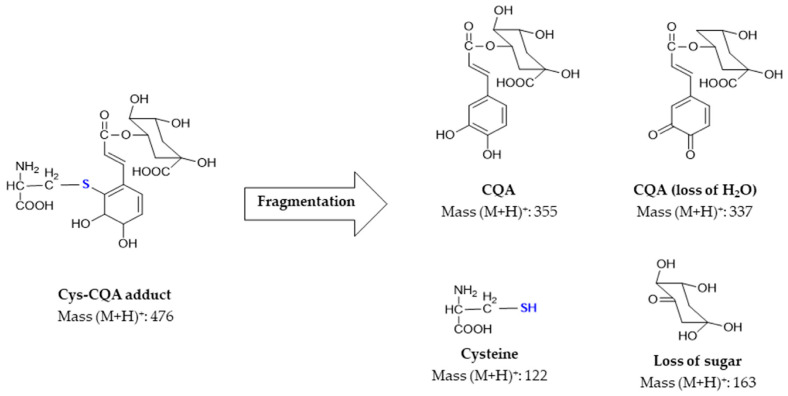
Proposed MS/MS fragmentation pattern of the *m*/*z* 476 in positive mode for the cysteine (Cys)-caffeoylquinic acid (CQA) adduct, with product masses tracked in the optimized method. [63].

The assembly state of protein subunits and macroscopic aggregation behavior directly influence the physical stability and processing characteristics of complex systems. Dynamic light scattering (DLS) and zeta potential analysis are widely used to monitor changes in the hydrodynamic radius and surface charge of a system, in order to assess whether physical fields effectively induce the disassembly of protein aggregation networks and changes in electrostatic repulsive forces [61]. Sodium dodecyl sulfate-polyacrylamide gel electrophoresis (SDS-PAGE) is a commonly used characterization method for studying subunit dissociation and cross-linking mechanisms. By comparing electrophoretic profiles under reducing and non-reducing conditions, it is possible to determine whether polyphenols bind to proteins via non-covalent bonds or induce covalent bonding between protein monomers. For morphological observations, a common approach involves the combined use of scanning electron microscopy (SEM), transmission electron microscopy (TEM), and atomic force microscopy (AFM). Among these, SEM and TEM can visually demonstrate the two-dimensional aggregation networks of complexes at the micro- and nanoscale, while AFM can further provide three-dimensional topography and roughness parameters of the complex surface, reflecting the degree of compactness or the unfolded conformation of macromolecular particles under the influence of physical fields. For the analysis of intact protein complexes or high-molecular-weight fragments, matrix-assisted laser desorption/ionization time-of-flight mass spectrometry (MALDI-TOF-MS) and electrospray ionization mass spectrometry (ESI-MS) are typically employed. These techniques can directly capture the characteristic mass shifts resulting from the covalent binding of protein monomers to polyphenols, thereby confirming the occurrence of the reaction and quantifying the amount of polyphenol bound. Furthermore, to enable precise analysis of microscopic binding sites, liquid chromatography-tandem mass spectrometry (LC-MS/MS) is widely used to analyze peptidic fragments following enzymatic digestion. Tracking specific *m*/*z* fragment ions via tandem mass spectrometry not only further validates the mechanism of the covalent addition reaction but also allows for the precise localization of cross-linking sites to specific amino acid residues within the peptide sequence (e.g., the thiol group of cysteine or the amino group of lysine) [62]. Research by Sagu et al. demonstrated that chlorogenic acid, after oxidation and a Michael addition reaction with the thiol group of cysteine, produces a characteristic mass shift in mass spectrometric characterization as shown in Figure 5. Specifically, after a covalent addition reaction with cysteine, a single molecule of chlorogenic acid forms a specific parent ion with a mass-to-charge ratio of 476 in the first-stage mass spectrometry. During the subsequent collision-induced dissociation (CID) process in the second-stage mass spectrometry, tracking this parent ion allows for the clear observation of fragment ions produced by its specific cleavage; by comparing these fragment peaks with fixed mass shifts in the mass spectrum, one can not only confirm the covalent binding between proteins and polyphenols but also determine that the binding site of the polyphenol is located on a specific thiol group [63].

## 4. Improvement of the Functional Properties of Protein–Polyphenol Complexes Through Physical Techniques

Furthermore, the combined action of physical techniques and polyphenols not only reshapes the molecular conformation of proteins and alters the physicochemical properties of the complex system at the microscopic level, but also influences the functional characteristics and application potential of protein–polyphenol complexes in food multiphase systems as shown in Table 2.

### 4.1. Interfacial Properties

Solubility is the physicochemical prerequisite for amphiphilic macromolecules to exhibit interfacial activity: physical field intervention not only effectively depolymerizes macroscopic protein aggregates and reduces the hydration particle size of the system, but also induces conformational unfolding, exposing more hydrophilic polar groups on the macromolecules, thereby enhancing the solubility of the complex in the aqueous phase [64]. The Emulsification Activity Index (EAI) and Emulsification Stability Index (ESI) are key indicators for evaluating protein emulsification capacity [65]. Studies have shown that after cold plasma treatment, the internal hydrophobic groups of oilseed rape protein are exposed, enhancing surface hydrophobicity and consequently increasing both the EAI and ESI. Upon binding with proanthocyanidins, this complex exhibits significant oil–water interfacial stability [66,67]. Furthermore, in gas–liquid interface systems, foaming capacity and foaming stability are similarly influenced by changes in protein structure. Ultrasonic treatment enables protocatechuic aldehyde molecules to form stable bonds with ovalbumin via hydrogen bonding. Results showed that the foaming capacity and foaming stability of ovalbumin increased by 27.5% and 5.5%, respectively. This may be attributed to the unfolding of the protein structure caused by ultrasonic treatment, which accelerated adsorption at the gas–liquid interface and the formation of a viscoelastic film, ultimately enhancing foaming stability. At the same time, the addition of polyphenols also enhanced the foaming capacity of the composite, thereby improving its overall foaming properties [68].

### 4.2. Solubility

Beyond serving as a prerequisite for interfacial activity, the improvement of aqueous solubility through physical field-modulated protein–polyphenol complexation is of significant practical importance for functional food formulations. Many bioactive polyphenols exhibit intrinsically poor water solubility, which limits their dispersion and bioavailability in aqueous food matrices. Physical field treatment addresses this limitation through dual mechanisms: on the protein side, cavitation or shear-induced unfolding exposes buried hydrophilic residues and polar groups, increasing the overall surface hydrophilicity of the complex; on the polyphenol side, binding to the physically unfolded protein confers enhanced dispersibility in water through the solubilizing effect of the protein carrier. Ultrasonic treatment of hemp seed protein-chlorogenic acid complexes increased system solubility by 38.7% and significantly enhanced emulsifying activity and emulsion stability indices [69]. Similarly, ultrasound-assisted complexation of rice bran protein with chlorogenic acid achieved a system solubility of 68%, attributed to the reduction in particle size and the exposure of hydrophilic groups induced by ultrasonic cavitation [70]. Dynamic high-pressure microfluidization (DHPM) treatment of soy protein isolate–rutin complexes also substantially improved solubility alongside increased surface charge and reduced particle size [35]. These findings demonstrate that physical field-assisted complexation serves as an effective strategy for enhancing the aqueous solubility of both proteins and polyphenols, thereby broadening their applicability in food systems.

### 4.3. Antioxidant Activity

Although polyphenols possess excellent antioxidant capacity, they are highly susceptible to degradation and inactivation in their free state due to environmental factors such as temperature and pH. However, certain non-thermal physical fields (e.g., ultrasound) can induce changes in protein structure, thereby exposing their hydrophobic regions and promoting binding with polyphenols, ultimately enhancing the antioxidant activity of the composite [25,71]. In antioxidant synergy, the overall antioxidant activity of the complex system is typically significantly higher than that of a physical mixture of individual components. This synergistic effect may stem from two aspects: on the one hand, after physical field treatment, amino acids within the protein are exposed to the solvent microenvironment, and these amino acids can act as antioxidants to participate in the radical quenching process; on the other hand, physical field treatment promotes the generation of more hydroxyl radicals, which improves the interaction between proteins and polyphenols, thereby enhancing the antioxidant activity of the complex. Beyond in vitro antioxidant capacity, the gastrointestinal stability and bioaccessibility of protein–polyphenol complexes are critical determinants of their potential health benefits. Experiments involving high-hydrostatic-pressure treatment of whey protein isolate and anthocyanin derivatives have demonstrated that appropriate high pressure not only promotes strong binding and enhances total antioxidant capacity but also substantially improves the digestive retention of active substances: after in vitro intestinal digestion, 38.17% of the bioactive compounds were retained, with sustained free radical scavenging activity superior to that of non-treated controls [72]. Recent studies further indicate that cold plasma treatment of soybean protein isolate with anthocyanin-rich extracts significantly enhances polyphenol bioaccessibility after simulated gastrointestinal digestion [73], and that complexation of beta-lactoglobulin with phlorizin can modulate the protein digestibility profile while promoting intestinal polyphenol uptake [74]. These findings suggest that physical field-assisted protein–polyphenol complexation serves a dual role: enhancing both the antioxidant potency and the digestive stability of the bioactive components. Nevertheless, direct in vivo evidence, including pharmacokinetic and bioavailability studies, remains limited and represents a critical direction for future research.

### 4.4. Antimicrobial Properties

Protein–polyphenol complexes have also demonstrated promising antimicrobial activity relevant to food safety and preservation. Polyphenols such as epigallocatechin gallate and chlorogenic acid possess inherent antimicrobial properties against a range of foodborne pathogens, attributed to their ability to disrupt bacterial cell membranes, chelate metal ions, and inhibit key microbial enzymes. When incorporated into physically modified protein matrices, the antimicrobial efficacy of these polyphenols can be further enhanced through improved chemical stability and sustained release. For instance, ultrasound-assisted incorporation of pomegranate polyphenol extract into tuna skin collagen-chitosan composite films resulted in significantly enhanced antibacterial activity against both Staphylococcus aureus and Escherichia coli, alongside improved mechanical properties and antioxidant activity [75]. These findings indicate that antimicrobial properties represent an additional functional dimension of protein–polyphenol complexes that merits further attention.

### 4.5. Gel Properties

Natural protein gels struggle to maintain good stability in complex external environments, which significantly limits their application in the food industry; this issue can be mitigated by adding polyphenols or through physical modification [76]. Studies have shown that, under the combined effects of high hydrostatic pressure and ultrasound, pea protein isolate undergoes significant denaturation and conformational unfolding when combined with anthocyanin-rich elderberry extract. Rheological and textural analyses indicate that the energy provided by the physical field forces the unfolded polypeptide chains to reorder and reassemble with the polyphenols, forming a three-dimensional network structure with a denser pore structure. This increase in microscopic cross-linking enhances the gel properties and stability of the composite; simultaneously, the dense physical network enhances the binding force on water molecules, improving the water-holding capacity of the composite gel, which is expected to increase its encapsulation efficiency for polyphenols [77]. Furthermore, in a composite system consisting of soy protein isolate fibrils and epigallocatechin gallate, the cavitation effect of ultrasound can effectively deconstruct the dense regions of the fibrils. This physical field-induced denaturation facilitates the formation of a denser hydrogen bond network between the surface of the soy protein isolate fibrils and epigallocatechin gallate. Under the synergistic action of this physical field and polyphenols, the formation of the composite gel is promoted, resulting in excellent gel properties [78].

**Table 2:** Improvement of functional properties of protein–polyphenol complexes and microscopic mechanisms by physical technology.

Technology	Complex Systems	Binding Type	Microscopic Mechanisms	Functional Improvements	References
Ultrasonic (US)	Rice bran protein–chlorogenic acid; hemp seed protein–chlorogenic acid; mung bean globulin–vitexin; whey protein–rutin	Non-covalent & covalent	Cavitation-induced unfolding exposes hydrophobic groups and reactive sites; promotes both non-covalent binding and radical-mediated covalent cross-linking	Solubility +38–68%; Emulsification Activity Index up to 126 m^2^/g; emulsion stability enhanced; surface hydrophobicity increased; particle size reduced	[6,24,69,70]
Dynamic High-Pressure Microjet (DHPM)	Pea protein–chlorogenic acid; soy protein isolate–rutin	Non-covalent	Instantaneous pressure drop and intense shear disrupt aggregates and induce conformational changes; exposes hydrophobic regions and free thiol groups	Solubility improved; emulsifying activity and rheological properties enhanced; extrusion properties improved; particle size reduced; surface charge increased	[35,36]
High-Pressure Homogenization (HPH)	Rice bran oil body protein–resveratrol; perch myofibrillar protein–EGCG	Non-covalent & covalent	Shear forces unfold aggregates and rearrange interfacial layers; covalent cross-linking between protein and polyphenol forms dense network structure	Surface hydrophobicity increased; emulsion stability enhanced; gel viscoelasticity and thixotropic recovery improved; cooking stability increased	[79,80]
Cold Plasma (CP)	β-Lactoglobulin–EGCG; green coffee protein–chlorogenic acid; quinoa protein–flavonoids; whey protein–EGCG	Non-covalent & covalent	High-energy electrons and free radicals promote covalent grafting; etching effect exposes reactive groups; oxidative modification increases surface polarity	Digestibility enhanced; antigenicity reduced; antimicrobial and antioxidant activity increased; solubility and foam stability improved; anti-nutritional factors decreased	[32,33,81,82]
Microwave (MW)	Soy protein–ferulic acid; whey protein–olive leaf extract	Non-covalent & covalent	Thermal energy drives molecular interactions and facilitates polyphenol release; promotes covalent cross-linking of protein side-chain groups	Gel texture, viscoelasticity, and water-holding capacity improved; antioxidant potential of edible coatings significantly enhanced	[83,84]
Pulsed Electric Field (PEF)	Asian clam myofibrillar protein–epicatechin gallate	Non-covalent	Irreversible electroporation enhances polyphenol infiltration into muscle tissue; electrostatic interactions induce conformational changes	Cooking loss and juice loss during cold storage reduced; preservation quality of aquatic muscle improved	[85]

## 5. Application of Physically Modulated Protein–Polyphenol Complex Systems in Food

**Figure 6:**
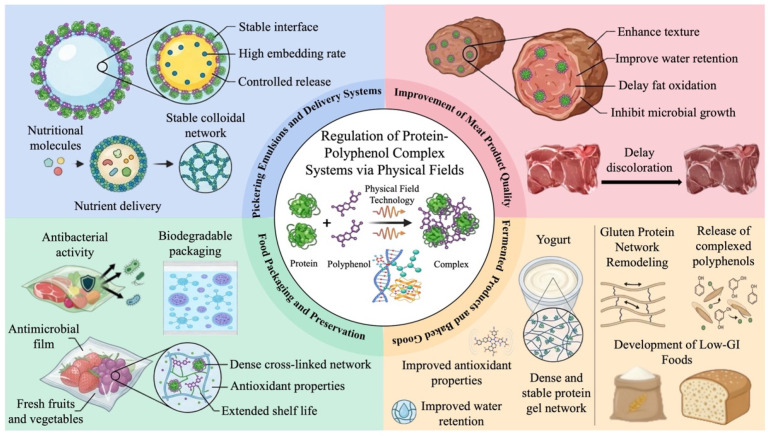
Application of protein–polyphenol complex system regulated by physical fields in food.

Physical field modulation endows protein–polyphenol complexes with superior interfacial properties, antioxidant activity, and gelling characteristics as shown in Figure 6. This structurally and functionally reengineered complex system is gradually emerging as a new type of clean-label ingredient, widely applied in multiple core areas of modern food processing. It effectively addresses the technical challenges of poor stability and susceptibility to degradation faced by traditional single ingredients in complex food matrices.

### 5.1. Pickering Emulsions and Carrier Systems

Protein nanocomposites formed through physical modification or polyphenol complexation have become ideal solid particle stabilizers for constructing high-internal-phase Pickering emulsions due to their suitable particle size and interfacial properties [86]. Studies have shown that the interfacial adsorption capacity and viscoelasticity of the ultrasonically treated egg yolk protein–epigallocatechin gallate complex system are improved. The high-internal-phase Pickering emulsion stabilized by this composite not only delays excessive lipid digestion but also improves the bioavailability and delivery efficiency of the encapsulated lipophilic bioactive substances [87]. Additionally, Dong et al. utilized the strong mechanical shear forces generated by dynamic high-pressure microjets to induce changes in the secondary and tertiary structures of pea protein, thereby enhancing its interfacial adsorption capacity. The emulsion prepared by ternary complexation of the modified protein, polyphenols, and polysaccharides exhibits excellent thermal stability, and the composite also demonstrates superior gelling properties and printability. This finding offers new possibilities for the application of pea protein in food 3D printing [36].

### 5.2. Food Packaging and Preservation

Untreated natural proteins typically exhibit physical defects such as low mechanical strength and high water vapor permeability after film formation. Through synergistic modification using physical fields and polyphenols, the three-dimensional cross-linking density of the film matrix can be increased, endowing it with functional properties such as antioxidant and antibacterial capabilities [88,89]. Research by Qu et al. found that, with the assistance of ultrasound, the combination of tuna skin collagen and pomegranate polyphenols—where the cavitation effect of ultrasound effectively disrupts protein aggregation and promotes non-covalent cross-linking between proteins and polyphenols—yields significant benefits. Compared to films not treated with ultrasound, films prepared from this modified system exhibited a 47.03% increase in tensile strength and a 24.16-fold increase in antioxidant capacity, demonstrating excellent film-forming toughness, antibacterial properties, and antioxidant capabilities, making them suitable for fresh food packaging [75]. Furthermore, dielectric barrier discharge (DBD) plasma technology has also demonstrated its ability to effectively modulate the film matrix properties of soy protein isolate and curcumin. Related studies indicate that after treatment with high-energy plasma particles, the thermal resistance of the composite system comprising soy protein isolate, curcumin, and carboxymethyl cellulose is improved. Concurrently, functional properties such as emulsification, gelling, and antioxidant activity are enhanced, demonstrating significant application potential in the field of microencapsulation of bioactive substances [90].

### 5.3. Quality Improvement of Meat Products

During high-temperature processing and refrigeration, proteins and lipids in meat products are prone to oxidative reactions, leading to textural deterioration and flavor loss [91]. Research by Li et al. indicates that the covalent binding efficiency between myosin and chlorogenic acid is significantly enhanced under combined microwave and ultrasonic treatment. Introducing the co-treated complex into a meat paste system not only improved the emulsifying properties of emulsion and addressed the inherent interfacial behavior defects of myosin but also established a robust antioxidant barrier through the phenolic hydroxyl groups on the surface of complex, thereby exhibiting excellent emulsifying and antioxidant performance [7]. Furthermore, cold plasma technology has demonstrated unique structure–activity advantages in meat preservation and the maintenance of gel texture. Studies have shown that the active components generated by cold plasma treatment can not only effectively inhibit spoilage microorganisms but also moderately oxidize the amino acid residues in the side chains of meat proteins. This synergizes with tea polyphenols to effectively suppress microbial proliferation in mutton and delay its discoloration. This technology not only significantly reduces the thiobarbituric acid reactive substances (TBARS, an indicator of lipid oxidation) in meat products but also mitigates the degree of protein oxidation, thereby helping to maintain the quality of mutton and extend its shelf life [92].

### 5.4. Fermented Dairy Products and Baked Goods

In the development of functional fermented dairy products, high-pressure treatment has demonstrated significant structure–activity regulation effects. Studies indicate that during the production of plant-based fermented dairy products rich in phenolic compounds (such as yogurt based on chickpeas, mung beans, or peas), untreated plant proteins are prone to phase separation in acidic fermentation environments [93]. However, by intervening with high-pressure treatment, the intense hydrostatic pressure overcomes intermolecular non-covalent forces, thereby inducing moderate denaturation of the plant proteins [94]. This conformational change not only allows the exposed protein side chains to form a more uniform and dense network structure with the phenolic compounds in the system but also significantly enhances the apparent viscosity and gelling properties of the yogurt. Compared to traditional thermal processing methods, high-pressure treatment not only effectively inhibits whey exudation during refrigeration without the addition of any hydrocolloid thickeners but also significantly improves the retention rate of total phenolic compounds and the overall antioxidant activity in yogurt [95].

In the field of baked goods, electromagnetic physical fields such as cold plasma and microwaves also play important barrier and strengthening roles. For whole wheat flour rich in phenolic compounds or doughs supplemented with exogenous polyphenols, reactive oxygen species generated by ambient-pressure cold plasma can mildly oxidize wheat gluten proteins, promoting the rearrangement of disulfide bonds. This physical-field-induced modification of protein structure not only significantly improves dough gas retention and final baking volume but also traps polyphenol molecules within a dense protein network, thereby substantially reducing the thermal degradation rate of heat-sensitive polyphenols during high-temperature baking [34]. Microwave treatment, on the other hand, reshapes the viscoelasticity of gluten proteins and promotes the release of bound phenols from the cereal matrix, providing a theoretical basis for the development of low-glycemic index (GI) baked goods that combine excellent texture with high free radical scavenging capacity.

## 6. Limitations and Perspectives

Although research on the regulation of protein–polyphenol interactions via physical fields has achieved certain results, further in-depth exploration is still required for their application in the food industry. Currently, research primarily focuses on the effects of individual physical fields, while studies on the synergistic mechanisms of different physical fields during modification are relatively scarce. Furthermore, future research should expand from single, purified protein–polyphenol systems to complex food matrix systems rich in polysaccharides or lipids, to thoroughly investigate the patterns of competitive adsorption and phase behavior evolution in multi-component systems. Concurrently, researchers should utilize animal models and multi-omics technologies to conduct in vivo bioavailability and toxicological assessments of the complexes formed after physical modification. Finally, to facilitate the translation of research findings into industrial applications, future research should focus on developing continuous, green preparation processes that combine multiple physical fields, integrating computational techniques to achieve precise control over the structure of the complexes, and actively promoting their application in areas such as targeted nutrient delivery, food preservation, and the development of functional foods.

## 7. Conclusions

As a green intervention method in modern food processing, physical modification offers new insights into regulating protein–polyphenol interactions and their structure–activity relationships. Non-thermal or thermal fields, such as ultrasound, ultra-high pressure, and cold plasma, can induce changes in protein conformation through different mechanisms, thereby influencing their interactions with polyphenols and ultimately optimizing the physicochemical properties and functional characteristics of the complexes. This lays the foundation for applications such as the development of stabilized delivery systems, functional foods, and active packaging materials.

## Figures and Tables

**Table 1:** Comparative analysis of physical technologies for protein modification: advantages, limitations, and industrial scalability.

Technology	Key Advantages	Key Limitations	Industrial Scalability	References
Ultrasound (US)	Low equipment cost; simple operation; effective cavitation-induced protein unfolding and enhanced polyphenol binding	Cavitation attenuation in large-volume reactors; localized heating; potential protein aggregation at high power densities	Widely studied at laboratory scale for protein modification; batch processing typical; scale-up challenged by non-uniform cavitation distribution in large reactors; industrial-scale ultrasonic systems are available for emulsification and extraction applications, with ongoing advances in reactor design to address scale-up challenges	[20,22,23,24,25,26,27,28]
High-Pressure Homogenization (HPH)	Continuous processing capability; efficient particle size reduction; uniform dispersion; well-established in dairy and emulsion industries	High capital investment; significant temperature rise during operation; limited to pumpable fluid systems	Highly scalable continuous process with established industrial infrastructure; widely adopted in dairy homogenization and emulsion processing; moderate-to-high energy requirements are offset by high throughput	[9,13,23,27]
Pulsed Electric Field (PEF)	Rapid, low-temperature treatment; minimal thermal damage to sensitive components; energy-efficient operation	Effectiveness depends on medium electrical conductivity; requires specific electrode geometry; primarily applicable to liquid systems	Scalable for continuous liquid flow processing; emerging industrial adoption for juice processing and liquid food preservation provides infrastructure precedent for protein applications	[19,23,29,30]
Microwave (MV)	Rapid volumetric heating; short treatment times (seconds to minutes); commercial equipment available at various scales	Non-uniform heating (hot spots); limited penetration depth in dense matrices; temperature control challenging	Established industrial microwave systems exist for food drying, heating, and sterilization; potential for continuous processing with appropriate reactor design	[10,14,23,27,31]
Cold Plasma (CP)	Ambient temperature; diverse reactive species generation (ROS, RNS); effective surface modification; minimal chemical residues	Reproducibility challenges; limited penetration into bulk liquids/solids; requires stable gas supply and discharge conditions	Atmospheric pressure operation advantageous for industrial integration; reactor design and scale-up actively developing; currently used in surface sterilization and food packaging applications at pilot scale	[11,12,15,23,27,32,33,34]
Dynamic High-Pressure Microfluidization (DHPM)	Extremely high shear forces achieve submicron particle sizes; effective aggregate dissociation; continuous operation	Very high capital and maintenance costs; chamber erosion from abrasive particles; limited to dilute suspensions; high energy demand	Suitable for high-value product processing where superior particle size reduction justifies cost; continuous operation compatible with industrial lines; current adoption limited to specialty applications due to equipment costs	[8,21,23,35,36]

## Data Availability

No new data were created or analyzed in this study. Data sharing is not applicable to this article.
